# Microtubule defects in mesenchymal stromal cells distinguish patients with Progressive Supranuclear Palsy

**DOI:** 10.1111/jcmm.13545

**Published:** 2018-03-04

**Authors:** Alessandra Maria Calogero, Mariele Viganò, Silvia Budelli, Daniela Galimberti, Chiara Fenoglio, Daniele Cartelli, Lorenza Lazzari, Petri Lehenkari, Margherita Canesi, Rosaria Giordano, Graziella Cappelletti, Gianni Pezzoli

**Affiliations:** ^1^ Department of Biosciences Università degli Studi di Milano Milan Italy; ^2^ Department of Services and Preventive Medicine Laboratory of Regenerative Medicine ‐ Cell Factory Fondazione IRCCS Ca' Granda Ospedale Maggiore Policlinico Milan Italy; ^3^ Parkinson Institute ASST G.Pini‐CTO, ex ICP Milan Italy; ^4^ Department of Physiopathology and Transplantation Dino Ferrari Center Neurodegenerative Disease Unit Fondazione Ca' Granda IRCCS Ospedale Policlinico University of Milan Milan Italy; ^5^ Department of Surgery and Anatomy Medical Research Center University of Oulu and University of Oulu Hospital Oulu Finland; ^6^ Center of Excellence on Neurodegenerative Diseases Università degli Studi di Milano Milan Italy

**Keywords:** bone marrow mesenchymal stromal cells, microtubules, neurodegeneration, Progressive Supranuclear Palsy

## Abstract

Progressive Supranuclear Palsy (PSP) is a rare neurodegenerative disease whose etiopathogenesis remains elusive. The intraneuronal accumulation of hyperphosphorylated Tau, a pivotal protein in regulating microtubules (MT), leads to include PSP into tauopathies. Pathological hallmarks are well known in neural cells but no word yet if PSP‐linked dysfunctions occur also in other cell types. We focused on bone marrow mesenchymal stromal cells (MSCs) that have recently gained attention for therapeutic interventions due to their anti‐inflammatory, antiapoptotic and trophic properties. Here, we aimed to investigate MSCs biology and to disclose if any disease‐linked defect occurs in this non‐neuronal compartment. First, we found that cells obtained from patients showed altered morphology and growth. Next, Western blotting analysis unravelled the imbalance in α‐tubulin post‐translational modifications and in MT stability. Interestingly, MT mass is significantly decreased in patient cells at baseline and differently changes overtime compared to controls, suggesting their inability to efficiently remodel MT cytoskeleton during ageing in culture. Thus, our results provide the first evidence that defects in MT regulation and stability occur and are detectable in a non‐neuronal compartment in patients with PSP. We suggest that MSCs could be a novel model system for unravelling cellular processes implicated in this neurodegenerative disorder.

## 1 INTRODUCTION

PSP, also known as Richardson‐Steele‐Olszewski syndrome, is a sporadic neurodegenerative disease described for the first time in 1963^1^ and for whom there are not available treatments to date. PSP results in severe disability, as it is characterized by frequent falls, supranuclear vertical gaze palsy, pseudobulbar palsy and rigidity of the neck.^2^ Thanks to its wide spectrum of clinical phenotypes, now this pathology has been recognized as a range of motor and behavioural syndromes^3^ and related to multiple pathological mechanisms.^4^ The disease is characterized by a neurodegenerative process that involves the basal ganglia, the prefrontal cortex and the cerebellum, with accumulation of tau protein, hence the classification as tauopathy.^5^ Tau is a MT‐binding protein encoded by the *MAPT* gene into 6 isoforms that are commonly referred to as 3R or 4R (with 3 or 4 MT‐binding domains, respectively). Tau binds to and stabilizes MTs, and promotes MT polymerization.^6^ The binding to MTs is regulated by phosphorylation of many residues; indeed, when hyperphosphorylated, tau detaches from MTs and accumulates forming neurofibrillary tangles (NFTs). All tauopathies are characterized by the presence of aggregates of abnormally phosphorylated tau protein, although the isoforms that aggregate vary.^7^ Both hyperphosphorylation and accumulation of 4R tau protein in neurons and glia, in basal ganglia and in the brain stem, are characteristic features of PSP.^8^ In PSP, the abnormal phosphorylation of tau triggers its detachment from MTs, mislocalization from the axon to dendrites and accumulation of still‐soluble “oligomers.”^9^

MTs are cytoskeletal polymers built up by α/β tubulin heterodimers, which participate in many cellular functions, such as maintenance of cell shape, cell migration and intracellular transport. MTs show a dynamic behaviour, switching between slow growth and rapid depolymerization^10^ and are finely regulated by the incorporation of specific α/β tubulin isotypes, by a plethora of MT‐binding proteins and by tubulin post‐translational modifications (PTMs).^11^, ^12^ Notably, α‐tubulin PTMs have been correlated with different MT subsets: tyrosinated MTs are the most dynamic ones, whereas acetylated or detyrosinated MTs are associated with more stable pools. The wide range of PTMs might, alone or in combination, generate chemical differences that are sufficient to confer cellular functions on MTs. Tubulin PTMs have important roles in regulating not only MT dynamics, but also motor traffic. Interestingly, defects in MT‐based transport in neurons, which are often linked to the accumulation of aggregated proteins, are typical of many neurodegenerative disorders, including Alzheimer's^13^ and Parkinson's (PD) diseases.^14^ In addition, it has been shown that MT stability and PTMs of tubulin are impaired in human fibroblasts derived from patients with PD.^15^

For PSP, there are currently no effective symptomatic or disease‐modifying treatments. In the last years, few clinical trials targeting mitochondria dysfunction, tau aggregation or MT stability have been performed or are ongoing.^16^ Besides other promising drugs, davunetide, which promotes MT stability, was effective as neuroprotective agent in a mouse model of tauopathy^17^ but it failed in a phase 2/3 clinical trial on patients with PSP,^18^ while TPI‐287, another MT stabilizer molecule, has recently entered a phase 1 clinical trial (Trial registration: ClinicalTrials.gov identifier NCT02133846). Among the ongoing trials, a therapy based on transplantation of undifferentiated human bone marrow MSCs has been proposed. MSCs are multipotent cells that can be isolated from many sources and whose therapeutic relevance is mostly due to their immunosuppressive and anti‐inflammatory properties.^19^, ^20^ Interestingly, beneficial effects of intravenous delivery of MSCs have been reported in rotenone‐treated mice, a PD model.^21^ Starting from encouraging pre‐clinical data, where MSCs show the ability to in vitro rescue 6‐hydroxydopamine‐damaged neural cell lines and to synthesize and secrete neurotrophines,^22^ we moved to a first pilot phase 1 study. In this trial, we had the dual aim to assess the safety of MSC therapy in a “first‐in‐man” context and the efficacy of autologous MSC treatment. Five patients have been treated in the open phase of our trial and at the end of this first step, we demonstrated the feasibility of autologous MSC administration in subjects with PSP and we recorded a clinical stabilization for at least 6 months (Trial registration ClinicalTrials.gov NCT01824121).^23^

To understand the real potential of patient‐derived MSCs, we performed in‐depth investigation of their biology. Specifically, we characterized the MT cytoskeleton of MSCs from patients affected by PSP, highlighting their characteristics in terms of MT stability and imbalance in α‐tubulin PTMs.

## 2 MATERIALS AND METHODS

### 2.1 Diagnostic criteria for PSP diagnosis

The criteria used for the diagnosis of PSP followed in this study are as follows: 1‐diagnosis of “probable Progressive Supranuclear Palsy‐Richardson's disease subtype” according to current diagnostic criteria,^2^, ^24^, ^25^ including akinetic‐rigid syndrome: gradually progressive disorder with age at onset of 40 years or later, vertical supranuclear palsy and prominent postural instability with falls within first year of disease onset; 2‐positive MRI for PSP criteria^26^; 3‐lack of response to chronic levodopa (at least 12‐month treatment).

### 2.2 Cell culture, subculture and cumulative population doublings

MSCs were obtained as previously reported in.^22^ Briefly, bone marrow was obtained after informed consent from aspiration of iliac crest and directly seeded in alpha‐modified Eagle's medium (alpha‐MEM; Macopharma, Mouvaux, France) supplemented with 10% high‐quality gamma‐irradiated foetal bovine serum (FBS) (Thermo Fisher Scientific, Waltham, MA, USA), at the concentration of 50 000 white blood cell (WBC)/cm^2^, at 37°C in a humidified atmosphere, 5% CO_2_. After 72 hours, non‐adherent cells were removed by washing with PBS (Macopharma) with complete medium change. On day 14, MSCs at P0 were detached using 0.04 mL/cm^2^ of TrypLE^™^ Select Enzyme (1X) (Thermo Fisher Scientific) and reseeded in the same culture conditions at the concentration of 4000 MSCs/cm^2^. Medium was replaced twice a week. MSCs were subcultured until they reached a plateau in the growth curve. Population doubling was calculated for each MSC lineage using the following equation: population doubling = log10(N)/log10(2); where N is the number of cells harvested at the end of the culture/the number of seeded cells. To define the expansion potential of cells, cumulative population doubling (CPD) was calculated by recording the cell counts and cellular dilution factor at each passage. Cell counting was performed by Burker chamber using Trypan Blue (Fluka, Buchs, Switzerland) to discriminate dead cells. For biochemical analyses, cells at passage 2 (P2) or passage 5 (P5) were seeded in 6‐well plates at a density of 5000 cells/cm^2^, whereas for immunofluorescence staining, 5700 cells/cm^2^ were seeded in BD Falcon CultureSlide (BD Bioscience, Jose, CA, USA). Cells were obtained from both controls (N = 6, age: 66.0 ± 1.18 years) and patients with PSP (N = 10, age: 66.1 ± 1.54 years) (Table 1).

**Table 1:** Demographic and clinical features of investigated subjects

	Healthy controls	PSP patients
Number of individuals (Male/Female)	6 (3/3)	10 (2/8)
Age^a^ (years): Median (range)	66 (62‐70)	66 (57‐75)
Mean (±SEM)	66.0 (±1.18)	66.1 (±1.54)
Disease onset age (years): Median (range)		62.5 (54‐69)
Mean (±SEM)		61.6 (±1.25)
Disease duration^a^ (years): Median (range)		4 (3‐7)
Mean (±SEM)		4.5 (±0.45)
Laterality onset: Left/Rigth/Bilateral		1/1/8
Exposure^b^: Positive/Negative/n.a.		0/10/0
Smoke: Positive/Negative/n.a.		3/6/1
Familiarity^c^: Positive/Negative/n.a.		3/5/2
PSP‐RS^a^: Median (range)		48 (35‐59)
UPDRS‐III^a^: Median (range)		39 (32‐51)
Supranuclear vertical gaze palsy^a^: Positive/Negative/n.a.		9/0/1
Pseudobulbar palsy^a^: Positive/Negative/n.a.		9/0/1
Rigidity of the neck^a^: Positive/Negative/n.a.		10/0/0
Pseudobulbar palsy (item 3 PSP‐RS): Median (range)		3 (1‐5)
Supranuclear vertical palsy (item 4 PSP‐RS): Median (range)		12 (6‐15)
Postural stability (item 30 UPDRS‐III): Median (range)		3 (2‐4)
Neck rigidity (item 22 UPDRS‐III): Median (range)		2 (1‐4)

n.a., not available; PSP‐RS, PSP Rating Scale; UPDRS‐III, Unified Parkinson's Disease Rating Scale, part III.

a At the time of BM collection.

b Professional exposure to toxic or mutagen substances.

c Familiarity for neurodegenerative disease.

### 2.3 Morphometric analysis

For morphometric analyses, 10 random images per well were captured using a Nikon Eclipse Ti‐S microscope (Nikon, Chiyoda, Japan), and analyses were made using ImageJ software (National Institute of Health, Bethesda, MD, USA) as previously described.^15^ For each cell, the maximum and minimum axes and the cell area were measured. The ratio between the maximum and minimum axis has been calculated. Only intact cells fully present into the image were considered.

### 2.4 Immunofluorescence

After 48 hours, cells were washed twice with PBS and fixed with methanol at −20°C. The samples were been treated with 5% BSA for 15 minutes at room temperature and incubated with the following primary antibodies: acetylated α‐tubulin mouse IgG (clone 6‐11 B‐1, SIGMA‐Aldrich, Darmstadt, Germany), tyrosinated α‐tubulin rat IgG (clone YL1/2; Abcam, Cambridge, UK), detyrosinated α‐tubulin rabbit IgG (ab48389; Abcam), in PBS, 1% BSA for 1 hour at 37°C. After washing twice with PBS, samples were incubated with Alexa Fluor^™^ 568 anti‐rat (Abcam), Alexa Fluor^™^ 488 anti‐mouse (Abcam) and Alexa Fluor^™^ anti‐rabbit 568 (Abcam) in PBS with 1% BSA for 45 minutes at 37°C. Nuclear staining was made with 4′,6‐Diamidino‐2‐phenylindole dihydrochloride (DAPI) (SIGMA‐Aldrich). The coverslips were mounted in Mowiol^®^ (Calbiochem, Darmstadt, Germany)—DABCO (SIGMA‐Aldrich) and examined with an Axiovert 200 M microscope (Carl Zeiss, Oberkochen, Germany).

### 2.5 Cell extracts

All cellular extracts were prepared in the presence of protease inhibitors (Protease Inhibitor Cocktail, P8340 SIGMA‐Aldrich). For preparation of whole‐cell extracts, cells were washed twice with PBS and scraped into SB1x (2% SDS, 10% glycerol, 5% β‐mercaptoethanol, 0.001% bromophenol blue and 62.5 mmol/L Tris, pH 6.8). Protein concentration was measured with Pierce BCA protein Assay Kit (Thermo Fisher Scientific). Equal amounts of each sample were separated by SDS‐PAGE. Cytosolic and cytoskeletal‐associated proteins were separated as previously reported.^27^ Briefly, cells were rinsed twice in PEM buffer (10 mmol/L EGTA, 1 mmol/L MgCl_2_, 88 mmol/L Pipes, pH 6.94), extracted for 10 minutes at room temperature with PEM buffer containing 0.1% Triton X‐100 and rinsed briefly in PEM buffer. The obtained Triton X‐100‐soluble fractions were diluted 3:1 with SB4x (soluble fraction). The insoluble material remaining attached to the dish was scraped into SB1x (insoluble fraction). Equal proportions of each fraction, representing proteins from the same number of cells, were separated by SDS‐PAGE.

### 2.6 Western blotting

Protein samples were separated by SDS‐PAGE and Western blotted onto PVDF membrane (Immobilon^®^‐P transfer membrane, IPVH00010; Immobilon^®^‐FL IPLL00010; Millipore, Darmstadt, Germany). Membranes were blocked with milk 5%, Tween 0.05% in Tris‐buffered saline (TBS) for 1 hour at room temperature. The membranes were probed over night at 4°C with the following primary antibodies: α‐tubulin mouse IgG (clone B‐5‐1‐2; SIGMA‐Aldrich), acetylated α‐tubulin mouse IgG (clone 6‐11 B‐1; SIGMA‐Aldrich), tyrosinated α‐tubulin rat IgG (clone YL1/2; Abcam), detyrosinated α‐tubulin rabbit IgG (ab48389; Abcam). The incubation with secondary antibodies for 1 hour at room temperature was performed using the following antibodies: anti‐mouse IgG HRP‐linked antibody (Cell Signaling Technology, Beverly, MA, USA), anti‐rabbit IgG HRP‐linked antibody (Pierce, Rockford, IL, USA), anti‐rat Alexa Fluor^™^ 568 (Abcam). Chemiluminescent signals were detected using Supersignal West Pico Chemiluminescent Substract kit (Pierce). Acquisition and quantification were performed by Chemidoc and Image Lab software (Bio‐Rad, Hercules, CA, USA).

### 2.7 Statistics

Statistical analysis was performed using STATISTICA (StatSoft Inc., Tulsa, OK, USA), and significant differences of PSP MSCs vs control MSCs, or between MSC‐A and MSC‐B, or between either of these and control MSCs were assessed by Student's *t*‐test or one‐way ANOVA with Tukey's post hoc test, respectively. For statistical analysis of CPD at single passage between PSP MSCs vs control MSCs, two‐way ANOVA with Sidak's multiple comparison test was performed. A *P*‐value <.05 was considered statistically significant.

### 2.8 Study approval

This study was approved by the Ethics Committee of Fondazione IRCCS Ca' Granda Ospedale Maggiore Policlinico (Authorization n.2795) and conforms to the declaration of Helsinki on ethical principles for medical research involving human subjects.

## 3 RESULTS

### 3.1 MSCs from patients with PSP show typical mesenchymal immunophenotyping but have late defects in proliferative capacity and altered morphology in culture

**Figure 1:**
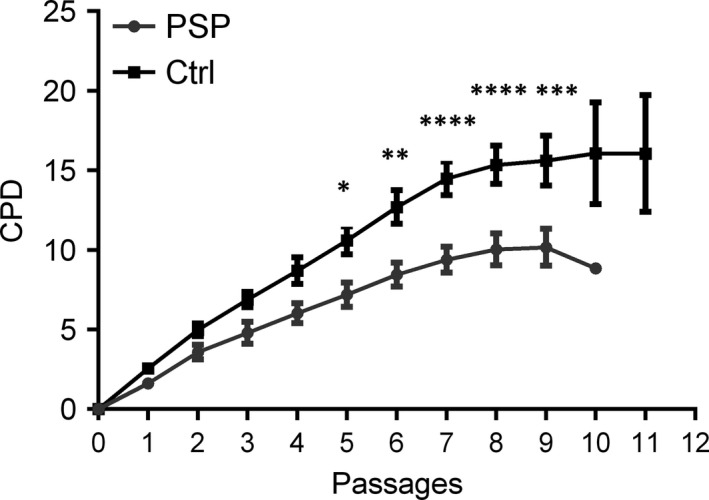
MSCs from patients with PSP show lower and shorter CPD compared to control cells. Growth kinetics of MSCs from healthy controls (Ctrl) and PSP patients (PSP) evaluated as CPD. All values are expressed as mean ± SEM. *P < .05, **P < .01, ***P < .001, ****P < .0001 PSP vs Ctrl at the same passage, according to ANOVA, Sidak's multiple comparison test. Ctrl: controls (N = 6); PSP: patients affected by PSP (N = 10)

To have a comprehensive characterization of MSCs from patients affected by PSP involved in this study (Table 1), we first evaluated the expression of many verified and postulated MSC cell surface antigens by flow cytometry on cultured cell lines at passage 2 (P2) in culture. In accordance with our previous data,^22^ their immunophenotypic profile confirmed that they are positive for CD90, CD105, CD73, CD13, CD146, PDGFR, ALP, and negative for CD45, CD34, CD14, CD3, CD40 and HLA‐DR‐like cells from age‐matched healthy controls (Table S1 and Figure S1). After that, we investigated the proliferative capacity of cultured MSCs by measuring the CPD (Figure 1). MSCs from healthy donors reached an average CPD value of 16 after 11 passages in culture, whereas the CPD of PSP cells reached the maximum value of 10.2 at passage 9, and then the curve started to decrease drastically. Moreover, the difference in growth kinetics between MSCs of patients with PSP and healthy controls became statistically significant starting from passage 5 (P5), showing that the in vitro proliferative capacity of MSCs from patients with PSP was significantly reduced.

**Figure 2:**
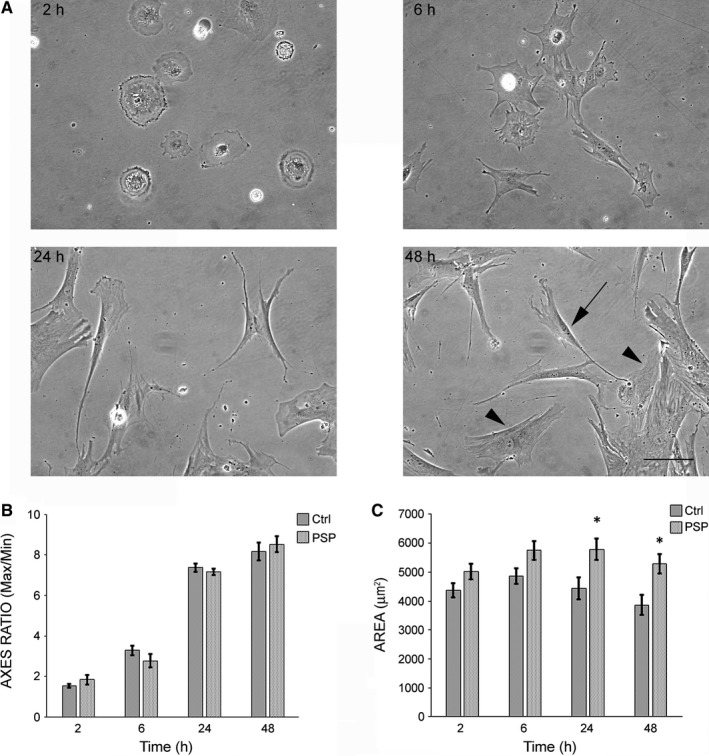
MSCs from patients with PSP show altered morphology compared to control cells. Representative phase contrast micrographs (A) of cultured human MSCs at different time‐points after seeding. Cells were evaluated just after they started to attach to the plastic (ie 2 and 6 hours) and then, when they have already established a stable connection with the culture surface (ie 24 and 48 hr). Arrowheads indicate flat or polygonal cells, arrow indicates fibroblast‐like cell. Scale bar: 100 μm. Morphometric analysis shows ratio between maximum and minimum cellular axes (B) or surface area (C) of MSCs of healthy controls (Ctrl) and PSP patients (PSP). *P < .05 PSP vs Ctrl according to Student's *t*‐test. All values are expressed as mean ± SEM. Ctrl: controls (N = 6), PSP, patients affected by PSP (N = 10)

Then, we analysed the morphology of cultured MSCs from patients with PSP and healthy controls at various time‐points after seeding (Figure 2). After 2 hours, cells adhered to the plastic substrate and were round. Six hours after seeding, cells started to lose their round shape and became elongated; moreover, many cellular projections were detectable. At later time‐points, cells clearly exhibited 2 different shapes: a fibroblast‐like morphology (arrow), or a large, flattened, polygonal or triangle shape (arrowhead), as previously reported.^28^ Despite the lack of any differences at first glance between healthy and PSP cells, detailed morphometric measurements, consisting in the evaluation of the ratio between maximum and minimum axis (Figure 2B) and the surface area (Figure 2C) of the cells, were performed. The measurements revealed increase in the maximum/minimum axis ratio overtime, as expected for elongating cells, but no actual differences between PSP and control cells. On the contrary, surface area was significantly increased in PSP cells compared to control cells at later time‐points after seeding. To exclude that these differences in cellular morphology could be masked by the large, flattened subpopulation of cells, we restricted morphometric analysis to the elongated and fibroblast‐like cells at later time‐points, and we obtained the same results as in the full population at 48 hours after seeding (Figure S2). Thus, taken together, these data show that MSCs from patients with PSP behave differently in culture as compared to healthy cells.

### 3.2 MSCs from patients with PSP show imbalance in α‐tubulin post‐translational modifications

**Figure 3:**
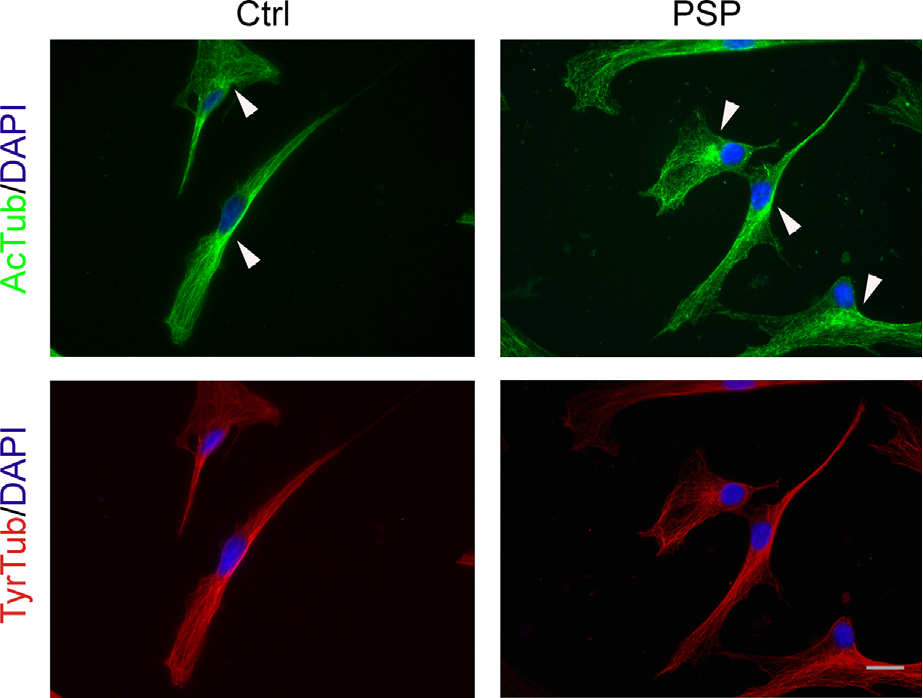
MSCs from patients with PSP show mild perinuclear enrichment of acetylated tubulin compared to control cells. Representative immunofluorescence of MSCs of patients affected by PSP (PSP) or healthy controls (Ctrl). Forty‐eight hours after seeding, cells were fixed and stained with anti‐acetylated (AcTub, green) or anti‐tyrosinated (TyrTub, red) α‐tubulin antibodies to detect MT cytoskeleton architecture and tubulin distribution. All cells were concurrently stained with DAPI (blue), to visualize the nucleus. Arrowheads indicate perinuclear enrichment. Scale bar: 20 μm

The observed changes in the morphology of PSP cells, compared to healthy controls, prompted us to investigate MT cytoskeleton in detail. First, we looked at MT architecture by immunodecorating cells at 48 hours after seeding. In particular, we focused on acetylated, detyrosinated and tyrosinated α‐tubulin localization. As reported in Figure 3, tyrosinated α‐tubulin was distributed throughout the cell both in control and PSP cells, being evident not only in the central region, where it accumulates, but also in the periphery. No evident differences were detectable between MSCs of patients affected by PSP and healthy controls. On the other hand, acetylated α‐tubulin localization was less widespread, being restricted mainly to the central region of the cell. Moreover, acetylated α‐tubulin accumulated around the nucleus, especially in PSP MSCs. In both control and PSP cells, acetylated α‐tubulin antibody immunodecorated short segments of MTs, while tyrosinated α‐tubulin antibody stains homogenously the MT network (Figure S3), as reported in other cell types.^29^ Detyrosinated α‐tubulin was not detectable in immunofluorescence assay (not shown).

**Figure 4:**
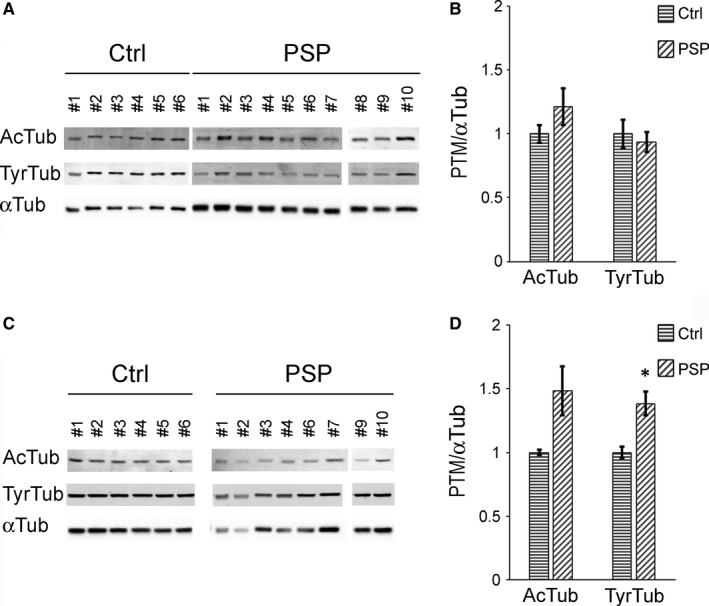
MSCs from patients affected by PSP show altered PTMs of α‐tubulin compared to control cells. Representative immunoblots (A, C) and densitometric analysis (B, D) of acetylated (AcTub), tyrosinated (TyrTub) or total (αTub) α‐tubulin protein expression, obtained from whole‐cell extracts of human MSCs from healthy controls (Ctrl) or PSP patients (PSP). Cells at early (P2; A and B) or later (P5; C and D) passages in culture were lysed and analysed to detect expression levels of AcTub, TyrTub or total αTub. Values of each α‐tubulin PTM were normalized on the level of αTub of the relative sample. Values are expressed as fold change on control level, error bars represent SEM. *P < .05 PSP vs Ctrl, according to Student's *t*‐test. Ctrl: controls (N = 6), PSP: patients affected by PSP (P2: N = 10, P5: N = 8)

Next, to investigate MT subsets in‐depth, we analysed the level of the various α‐tubulin PTMs in whole‐cell lysates of MSCs of healthy controls and patients with PSP by Western blotting and densitometric analysis (Figure 4). We analysed cells not only at P2 but also at P5 in culture, when cells started to proliferate differently compared to controls (Figure 1).

As reported in Figure 4B, at the earliest time‐point, PSP cells contained as much acetylated and tyrosinated α‐tubulin as cells of healthy controls. Detyrosinated α‐tubulin was not detectable by Western blotting analysis too (not shown). The lack of detyrosinated α‐tubulin in both PSP and controls may be reasonably due to the particular undifferentiated nature of stromal cells, as this modified form of α‐tubulin is associated with one of the most stable pools of MTs. The analysis performed at P5 showed enrichment of acetylated α‐tubulin in PSP cells and, in addition, a significant increase in tyrosinated α‐tubulin compared to control cells (Figure 4D).

Based on these data, we can conclude that the imbalance in α‐tubulin PTMs becomes evident overtime in PSP cells. As PTMs on α‐tubulin are not merely associated with pools of MT with different stability, but they are also involved in the maintenance of proper MT functions, their imbalance could have a strong impact on many cellular processes.

### 3.3 MSCs from patients with PSP show altered microtubule stability compared to controls

**Figure 5:**
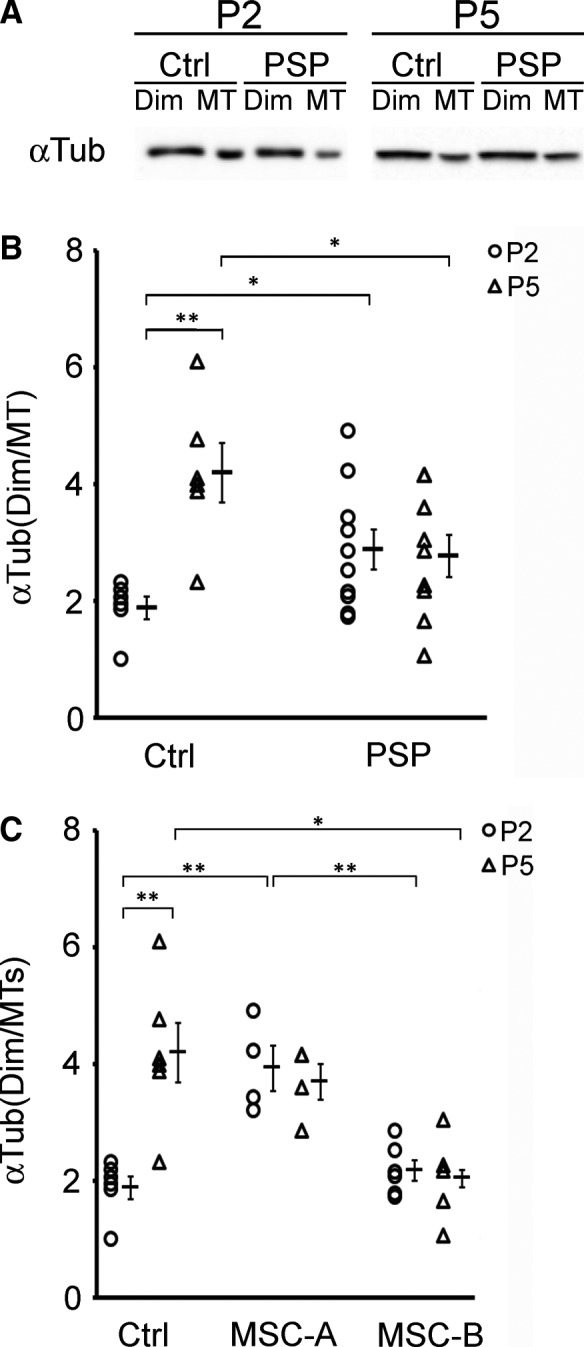
Impairment of MT stability is shared by MSCs from patients with PSP. (A) Representative immunoblot of α‐tubulin (αTub) levels in soluble (Dim) or insoluble (MT) fractions of MSCs of healthy controls (Ctrl) or PSP‐affected patients (PSP) groups, at early (P2) or later (P5) passages in culture. (B) To evaluate MT mass, densitometric analysis of αTub levels of each fraction was performed and the obtained values, expressed as Dim/MT ratio, are shown graphically. Circles and triangles represent values of single controls or patients, while the short horizontal lines represent the mean values within each group. Error bars = SEM. Circles = data obtained at passage 2 (P2) in culture; triangles = data obtained at passage 5 (P5) in culture. *P < .05, **P < .005 according to Student's *t*‐test. (C) Densitometric analysis of αTub levels, expressed as Dim/MT ratio, of controls and PSP‐A subgroup or PSP‐B subgroup of patients. Circles = P2; triangles = P5. *P < .05, **P < .005 according to ANOVA, Tukey's post hoc test. Ctrl: controls (N = 6), PSP: patients affected by PSP (P2: N = 10, P5: N = 9), MSC‐A: subgroup A of patients affected by PSP (N = 4), MSC‐B: subgroup B of patients affected by PSP (P2: N = 6, P5: N = 5)

To verify whether the observed differences in tubulin PTMs between MSCs from patients with PSP and healthy controls correlate with altered MT stability, we investigated MT mass. By Western blotting and densitometric analyses (Figure 5), we evaluated the amount of α‐tubulin in the soluble fraction (ie the dimeric pool) and in the insoluble fraction (ie the polymerized MT fraction) of PSP and control cells. As reported in Figure 5B, at early passage (P2), the ratio between free α‐tubulin vs α‐tubulin incorporated into MTs (Dim/MT) was significantly increased in PSP cells compared to cells from healthy controls. These data mean that MSCs from patients with PSP undergo MT destabilization, suggesting that defects in the polymerization or depolymerization of MTs occur. On the contrary, at later passage (P5), a higher Dim/MT ratio was found in control cells compared to patients with PSP and, interestingly, compared to P2 controls. Although MT stability remains unmodified in patients with PSP, the behaviour of control cells changes overtime. This could indicate that cells isolated from patients are less prone to undergo MT rearrangements.

Investigating the distribution of Dim/MT ratio values among the patients, we observed that MSCs from the PSP group could be further divided into 2 subgroups: MSC‐A (including cells obtained from PSP#1, #3, #7, #10 patients) and MSC‐B (including cells from PSP #2, #4, #5, #6, #8, #9 patients) as listed in Table S2. As reported in Figure 5C, at early passage in culture, MSC‐B subgroup showed a Dim/MT ratio similar to the control group, while MSC‐A showed a significant increase in Dim/MT ratio compared to both controls and MSC‐B subgroup (about 2 folds). Thus, the analysis of MT mass disclosed the presence of 2 subgroups of PSP MSCs, one of which (MSC‐A) was characterized by early MT destabilization, whereas the other one (MSC‐B) seemed to be similar to the control group. On the contrary, looking at later passages in culture, we observed that MSC‐A cells did not show any change, whereas MSC‐B cells showed a significant decrease in Dim/MT ratio when compared to the control cells.

The assessment of the time course (P2 vs P5) indicated that Dim/MT ratio does not change overtime in both PSP MSC subgroups. Thus, the fact that healthy controls were characterized by an increase in Dim/MT ratio overtime indicates that culture conditions induce MT destabilization per se in control cells. Conversely, both MSC‐A and MSC‐B cells did not undergo these changes, suggesting that they are less sensitive to the potential stress induced by culturing. The 2 subgroups did not differ for any of the other investigated parameters (Figure S4).

All together, these data clearly reveal that MSCs from patients with PSP are characterized by defects in MT stability regulation compared to control cells. This suggests that polymerization (or depolymerization) of MTs may be altered in cells of patients affected by this neurodegenerative disorder. Although at early time‐points, a subgroup of PSP MSCs (ie MSC‐B) showed a similar distribution of α‐tubulin between free and polymerized pools compared to control cells, we can conclude that the lack of change overtime observed in both the 2 PSP MSC subgroups clearly supports the concept that defects in MT stability distinguish PSP MSCs.

## 4 DISCUSSION

PSP is a rare neurodegenerative disorder that affects various areas of the brain, including basal ganglia, brainstem, cerebral cortex, dentate nucleus and spinal cord regions, and that can affect not only neurons, but also glial cells.^30^, ^31^ The mechanism(s) that can lead to PSP are not yet understood, as well as its causes, that include both environmental (alkaloids) and genetic (*MAPT* mutations, haplotype) factors.^4^ The fact that an important hallmark of the disease is the presence of abnormal cerebral aggregates of tau protein, a MT‐binding protein, suggests that MT dysfunction may be an important element of the disease. Obviously, the difficulty of working on living cells obtained from the affected brain regions of patients with PSP limits the possibility to investigate the cellular mechanisms underlying the disease and prompts the search for reliable human cellular models of the disease. Here, we investigated MT system in MSCs of patients with PSP and disclosed significant defects in comparison with healthy controls. Showing the imbalance of α‐tubulin PTMs and the impairment of MT stability, this study demonstrates for the first time that MT dysfunction distinguishes PSP MSCs. It also highlights that this signature is detectable in non‐neural and undifferentiated cells such as MSCs.

From a general point of view, MTs are key elements of all the cells, being responsible for the maintenance of morphology, organelle trafficking and intracellular transport processes, but their importance is highlighted in cell types with a complex morphological architecture such as neurons or oligodendrocytes,^32^, ^33^ which are both involved in PSP disease. A growing amount of evidence supports the concept that MT loss, altered MT dynamics and axonal transport defects are linked to neurodegenerative processes,^34^, ^35^ and that MT stability could be a potential therapeutic target.^36^, ^37^^.^We investigated MT system in MSCs moving from the analyses of α‐tubulin PTMs to the evaluation of MT mass. Tubulin PTMs have the potential to generate chemical differences defining a “tubulin code” on MTs^38^, ^39^, ^40^ and, thus, complex molecular signals sufficient to control the interaction of MTs with several proteins, including MT‐interacting proteins (MIPs) and motor proteins. On this basis, they are emerging as crucial controllers of MT properties and functions beyond the well‐known role as markers of MTs with different stability. Firstly, we found that detyrosinated α‐tubulin is undetectable in MSCs. This finding supports the concept that their MT cytoskeleton is highly dynamic and lacks long‐lived detyrosinated MTs, as expected in cycling cells. In addition, the levels of tyrosinated α‐tubulin are higher in late passage MSCs derived from patients with PSP than in those derived from healthy controls. This could suggest that MT cytoskeleton is more dynamic in patients, tyrosinated α‐tubulin being found in the unpolymerized pool of tubulin and in neo‐synthetized MTs, or that cytoskeleton changes its ability to bind MIPs. Indeed, the C‐terminal tyrosine on α‐tubulin can act as a binary ON/OFF switch for the recruitment of MT dynamics regulators, such as kinesin 13 MCAK, which preferentially depolymerizes tyrosinated MTs.^41^ In addition, the C‐terminal tyrosine is also required for the recruitment of MT plus‐end‐interacting proteins, such as cytoplasmatic linker protein‐170 (CLIP170).^42^ Therefore, the increasing amount of tyrosinated α‐tubulin that we observed in PSP cells could, in turn, alter the interactions with various MIPs.

Here, we found that MSCs from patients with PSP are characterized by the loss of MT mass at baseline. The ratio between free tubulin and MTs is altered in PSP cells, tubulin being shifted towards the unpolymerized pool. This suggests that destabilization of MTs occurs in PSP cells at baseline. Interestingly, MT mass does not change overtime in cultured PSP cells, whereas it decreases in control cells, and this can be read as the inability of patient cells to efficiently remodel MT cytoskeleton during ageing in culture. Next, we found that PSP MSCs are heterogeneous in terms of initial MT mass leading us to identify 2 subgroups. However, this does not weaken the concept that MT mass regulation/dynamics differ between controls and PSP‐affected patients. Indeed, although one of the MSCs subgroups resembles controls in terms of initial MT destabilization, they are both characterized by the absence of changes overtime. This is a very intriguing point as the detailed clinical evaluation of patients, whose cells have been included into the 2 subgroups, does not uncover any difference (Table S2). In addition, deeper investigating our patients for *MAPT* gene haplotype, we found that only one (PSP#3, MSC‐A subgroup) shows an allele of H2 haplotype, thus indicating that there is not a correlation between H1/H2 haplotype distribution and Dim/MT ratio in any of the 2 MSCs subgroups (Table S3). In conclusion, the differences highlighted in the MSCs of the 2 subgroups seem to be not linked with PSP genetic factors or with the clinical features of patients, but could be the signature that might have the potential to drive more specific and in‐depth stratification of patients.

Speculating about the impact of MT defects on cell functionality, many are the MT‐dependent events in all cell types other than neurons and oligodendrocytes. Among them, intracellular trafficking is emerging as a key regulator of diverse processes such as cell division, migration and secretion, thus confirming that MT dysfunction could be really detrimental for cells. Defects in the secretion pathways could be linked to many neurodegenerative disorders as suggested by recent evidences reporting changes in the serum levels of modulatory factors in patients affected by PD,^43^ MSA^44^ and AD.^45^ Notably, specific properties of MSCs cells include the secretion of a set of factors/molecules to the extracellular space, the so‐called secretome, whose crucial role in anti‐inflammatory response makes them a promising therapeutic tool.^46^ Here, our detailed analysis of MSCs of patients with PSP unravels MT defects, reduced proliferative capacity and predisposition to ageing in culture conditions. Whether these defects can affect their secretory profile and therefore to their paracrine reparative effects is still under investigation, but the clinical use of early passage MSCs is strongly recommended when autologous MSCs are tested in phase 1/2 clinical trials. Notably, also in the clinical trial performed by our group based on the autologous use of MSCs in patients with PSP, the administered cellular products were composed by maximum passage 2 MSCs.^22^

Peripheral tissues are a source of human living cells, and in the last few years, they have become reliable models for the identification of molecular alterations and possible therapeutic targets in neurodegenerative disorders. For example, skin primary fibroblasts are considered a good model system for PD^47^ and, interestingly, we have previously reported that fibroblasts of patients with PD are characterized by changes in MT mass compared to controls.^15^ Focusing on MSCs, they are relatively easy to obtain, to maintain and to expand in culture. The relevance of studying this cell type for unravelling defects linked to neuronal disorders is completely unexplored. For this reason, our detailed analysis of MT system is a starting point for moving to neuronal models obtained from patients that are actually not available (eg iPSC‐derived neurons). Beyond being a potential model for studying PSP‐linked dysfunction, MCSc could be used for drug screening. Indeed, Polioudaki et al^48^ showed that taxol and nocodazole, 2 well‐known MT‐interacting drugs, can induce moderate and reversible damage to MSCs of healthy donors. As we found that control and PSP‐derived MSCs are characterized by differences in MT system, it will be intriguing to evaluate if they differently respond to anti‐MT drugs. Thus, MSCs are also a very precious and promising tool for personalization of drug screening and therapies.

In conclusion, for the first time, our study unravels the characteristics of MT cytoskeleton in MSCs from patients affected by PSP, a rare neurodegenerative disorder, and demonstrates that these cells differ from healthy controls in terms of MT stability, α‐tubulin PTMs, cell morphology and growth. This is in accordance with a very recent study by a part of our group showing that mitochondrial dysfunction occurs in MSCs from patients with PSP.^49^ This suggests that the mechanisms leading to PSP might also affect undifferentiated non‐neural cells. Our results pave the way to the experimental use of alternative cellular models as in vitro system for deciphering the intracellular mechanisms of PSP and identifying novel pharmacological targets, thus ultimately helping in finding new therapeutic approaches to PSP as well as other still orphan neurologic diseases.

## CONFLICT OF INTEREST

The authors have declared that no conflict of interest exists.

## AUTHORS' CONTRIBUTIONS

A.M.C., D.C., L.L., M.C., R.G., G.C. and G.P. conceived the study and participated in its design. R.G, M.C. and G.P. recruited patients and collected clinical data. M.V. and S.B. isolated, cultured and characterized MSC. A.M.C. performed morphometry and immunofluorescence assays, analysed MSCs' extracts and performed MT's profiling assays. D.G. and C.F. performed genotypic analysis. P.L. provided and characterized control samples. A.M.C. and G.C. wrote the manuscript with critical contributions from all the authors. All the authors participated in data analysis and interpretation, reviewed and approved the final manuscript.
