# Supplementary material for: Microtubule defects in mesenchymal stromal cells distinguish patients with Progressive Supranuclear Palsy
Source: J Cell Mol Med. 2018 Mar 4;22(5):2670–9. doi: 10.1111/jcmm.13545 (PMC5908108; doi:10.1111/jcmm.13545)
Supplement: Supplementary file 1 [file JCMM-22-2670-s001.pdf]

Microtubule defects in mesenchymal stromal cells distinguish Progressive Supranuclear Palsy patients

Alessandra Maria Calogero[1§], Mariele Viganò [2§], Silvia Budelli[2, 5], Daniela Galimberti[3], Chiara Fenoglio[3], Daniele Cartelli[1], Lorenza Lazzari[2], Petri Lehenkari[4], Margherita Canesi[5], Rosaria Giordano[2], Graziella Cappelletti[1,6 #,*] and Gianni Pezzoli[5#]

[1] Department of Biosciences, Università degli Studi di Milano, Milan, Italy

[2] Department of Services and Preventive Medicine, Laboratory of Regenerative Medicine - Cell Factory, Fondazione IRCCS Ca' Granda Ospedale Maggiore Policlinico, Milan, Italy

[3] Department. of Physiopathology and Transplantation, University of Milan, Dino Ferrari Center, Neurodegenerative Disease Unit, Fondazione Ca' Granda, IRCCS Ospedale Policlinico, Milan, Italy

[4] Department of Surgery and Anatomy, Medical Research Center University of Oulu and University of Oulu Hospital, Oulu, Finland

[5] Parkinson Institute, ASST G.Pini-CTO, ex ICP, Milan, Italy

[6] Center of Excellence on Neurodegenerative Diseases, Università degli Studi di Milano, Milan, Italy

§ these authors contributed equally to the work.

# co-last authors

* corresponding author: Graziella Cappelletti, Department of Biosciences, Università degli Studi di Milano, Via Celoria 26, Milano, 20133, Italy. Tel: +39 0250314752; Fax: +30 0250315044; Email: graziella.cappelletti@unimi.it

SUPPLEMENTARY MATERIAL AND METHODS

Flow cytometry analysis

MSCs from age-matched healthy donors and PSP patients were extensively characterized by flow cytometry at P2. Cells were washed in PBS and incubated in the dark for 20 minutes at RT with the following mouse anti-human antibodies: CD3 FITC (Becton Dickinson - BD, San Jose, CA, USA), CD13 PE (Beckman Coulter - BC, Brea, CA, USA), CD14 PerCP-Cy5.5 (BD), CD34 PE (BD), CD40 FITC (BD), CD45 APC-H7 (BD), CD90 PE-Cy7 (BD), CD73 APC (BD), CD105 PerCP-Cy5.5 (BD), CD146 PE-Cy7 (BD), HLA-DR APC (BD), Platelet-Derived Growth Factor Receptor Beta (PDGFRß) PE (BD) and Alkaline Phosphatase (ALP) FITC (BD). No stained cells were used as negative controls under the same conditions. After staining, the cells were washed once with PBS. At least 10,000 events were acquired with FACS CANTOII flow cytometer (BD) and data were analyzed using the FACSDiva analysis software.

Genetic analysis of *MAPT* haplotype

MAPT exons 1 and 9-13 were sequenced as previously described [1] . Briefly, each PCR was carried out in 50 µl total volume containing 25 ng genomic DNA, 12.5 pmol of each specific primer, 0.6 µM of each dATP, dTTP, dCTP, and dGTP, 1U Taq DNA polymerase (Applied Biosystems, ABI). Fragments were then purified using the ExoSAP-IT® Kit (usb, USA), according to instructions of the manufacturer, and then direct sequence was performed with an ABI PRISM 3130 gene analyzer (ABI).

The tagging *MAPT* H1/H2 SNP rs1800547 [2] was genotyped using TaqMan technology by QuantStudio 12K Flex System (Applied Biosystems, Lifetech, USA) according to the procedure of the manufacturer.

REFERENCES

1. **Villa C, Ghezzi L, Pietroboni AM, *et al.*** A Novel MAPT Mutation Associated with the Clinical Phenotype of Progressive Nonfluent Aphasia. *J Alzheimer's Dis*. 2011;26:19-26.

2. **Tobin JE, Latourelle JC, Lew MF, *et al.*** Haplotypes and gene expression implicate the MAPT region for Parkinson disease: the GenePD Study. *Neurology*. 2008;71:28-34.

SUPPLEMENTARY TABLES

Table S1: Flow cytometry analysis of expanded MSCs

Surface Antigen	**Mean ± SD (%)[a]**	
	**Ctrl**	**PSP**
CD90+	99.5 (±0.6)	99.2 (±0.6)
CD105+	97.5 (±4)	98.2 (±1.4)
CD73+	99.5 (±0.7)	95.3 (± 5.0)
CD13+	97.8 (±0.8)	96.5 (± 3.3)
CD146+	94.2 (±8.1)	91.8 (±5.9)
PDGFRβ+	94.4 (±8.2)	92.9 (±7.2)
ALP+	92.5 (±2.1)	91.3 (±5.1)
CD45+	1 (±0.3)	0.2 (±0.1)
CD34+	0.2 (±0.1)	0.1 (±0.1)
CD14+	0.4 (±0.6)	0.1 (±0.1)
CD3+	0.0 (±0.0)	0.3 (±0.3)
CD40+	0.1 (±0.1)	0.2 (±0.2)
HLA-DR+	0.9 (±0.9)	0.3 (±0.3)

[a]= percentage of cells positive for surface markers expressed as mean and standard deviation (N=5)

Table S2: Demographic and clinical characteristics of patients whose cells have been investigated

MSCs Subgroup	Patient ID	Gender	Onset age (years)	Disease duration [a] (years)	Laterality onset	Exposure [b]	Familiarity [c]	Smoke	PSP-RS	UPDRS-III	Pseudobulbar Palsy (item 3 PSP-RS)	Supranuclear Vertical Palsy (item 4 PSP-RS)	Postural Stability (item 30 UPDRS-III)	Neck Rigidity (item 22 UPDRS-III)
**MSC-A**	PSP#1	F	63	3	Bilateral	Neg	AD	Pos	48	32	3	14	2	1
	PSP#3	F	54	3	Bilateral	Neg	Neg	Pos	49	39	1	10	4	1
	PSP#7	F	63	5	Left	Neg	Neg	Neg	58	46	5	12	4	2
	PSP#10	F	63	4	Bilateral	Neg	n.a.	Neg	47	39	4	15	3	1
	Median		63	3.5					48.5	39	3.5	13	3.5	1
	(range)		(54-63)	(3-5)					(47-58)	(32-46)	(1-5)	(10-15)	(2-4)	(1-2)
**MSC-B**	PSP#2	F	64	7	Bilateral	Neg	n.a.	Neg	59	51	4	10	3	4
	PSP#4	F	62	4	Bilateral	Neg	AD	Neg	52	49	5	12	3	4
	PSP#5	F	69	6	Right	Neg	Neg	Neg	46	35	3	12	2	2
	PSP#6	F	59	6	Bilateral	Neg	PD	Neg	40	39	2	14	3	2
	PSP#8	M	59	3	Bilateral	Neg	Neg	n.a.	n.a.	50	n.a.	n.a.	3	3
	PSP#9	M	60	4	Bilateral	Neg	Neg	Pos	39	34	3	6	3	1
	Median		61	5					46	44	3	12	3	2.5
	(Range)		(59-69)	(3-7)					(39-59)	(34-51)	(2-5)	(6-14)	(2-3)	(1-4)

[a]=at time of BM collection; [b] = Professional exposure to toxic or mutagen substances; [c]= familiarity for neurodegenerative disease; AD = Alzheimer's disease; PD = Parkinson's disease; n.a. = not available; PSP-RS= PSP Rating Scale; UPDRS-III= Unified Parkinson's disease Rating Scale, part III.

Table S3: *MAPT* haplotype analysis of investigated patients

MSCs subgroup	Patient ID	Genotype rs1800547 A/G	Haplotype
MSC-A	PSP#1	A/A	H1/H1
	PSP#3	A/G	H1/H2
	PSP#7	A/A	H1/H1
	PSP#10	A/A	H1/H1
MSC-B	PSP#2	A/A	H1/H1
	PSP#4	A/A	H1/H1
	PSP#5	A/A	H1/H1
	PSP#6	n.a.	n.a.
	PSP#8	A/A	H1/H1
	PSP#9	A/A	H1/H1

n.a. = not available

SUPPLEMENTARY FIGURES AND FIGURE LEGENDS

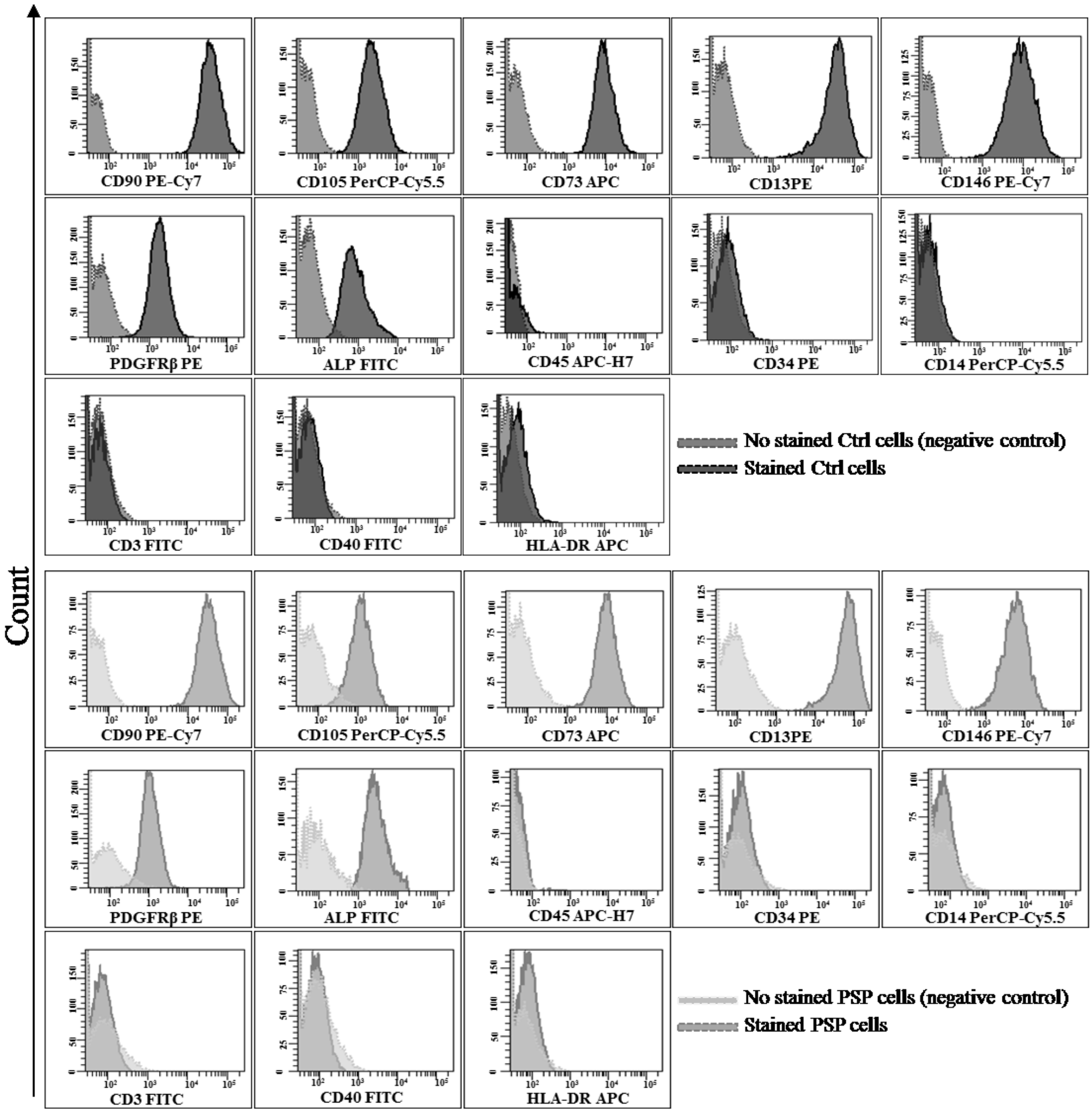

Fig. S1 Immunophenotypic profile of MSCs from PSP patients and healthy control

Representative MSC immunophenotypic profile by flow cytometry at passage 2 of healthy controls (Ctrl) and PSP patient (PSP) cells. Fluorescence intensity is displayed on the X-axis and the number of events (Count) in each fluorescence channel is displayed on the Y-axis. Specific histogram of fluorescence for each marker was overlaid with its negative control (no stained cells).

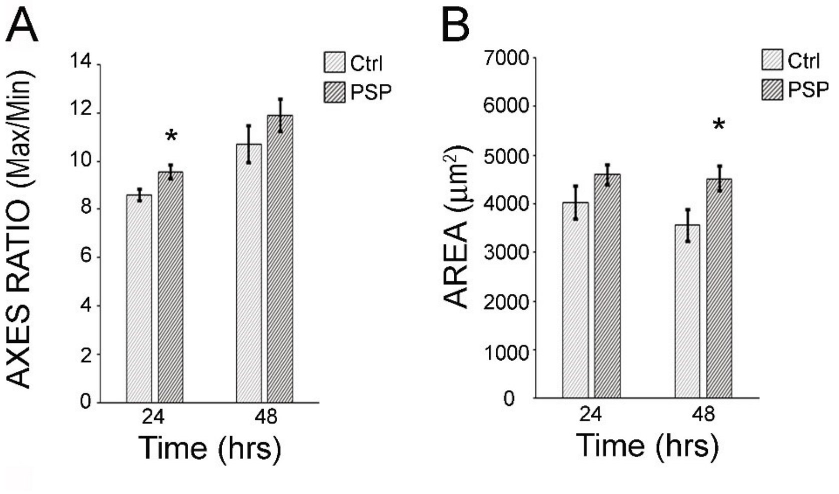

Fig S2: Morphometric analysis of fibroblast-like subpopulation of MSCs at 24 or 48 hours after seeding

Morphometric analysis showing ratio between maximum and minimum cellular axes (A) or surface area (B) of MSCs of healthy controls (Ctrl) compared to PSP-affected patients (PSP). *$p<0.05$ according to Student's t-test. All values are expressed as mean ± SEM. Ctrl: controls (N=6), PSP: patients affected by PSP (N=10).

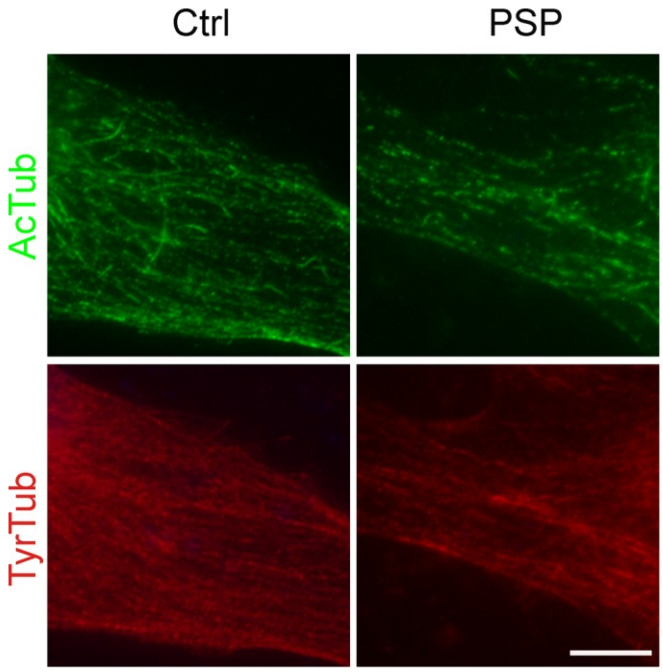

Fig. S3: Acetylated and tyrosinated microtubules show different staining patterns in MSCs

High magnification of representative immunofluorescence of MSCs of patients affected by PSP (PSP) or healthy controls (Ctrl) following staining for anti-acetylated (AcTub, green) or anti-tyrosinated (TyrTub, red) tubulin. Anti-acetylated α-tubulin antibodies immunodecorate short segments of MTs, while anti-tyrosinated α-tubulin staining is more homogeneous over the MT network. Scale bar: 10 μm.

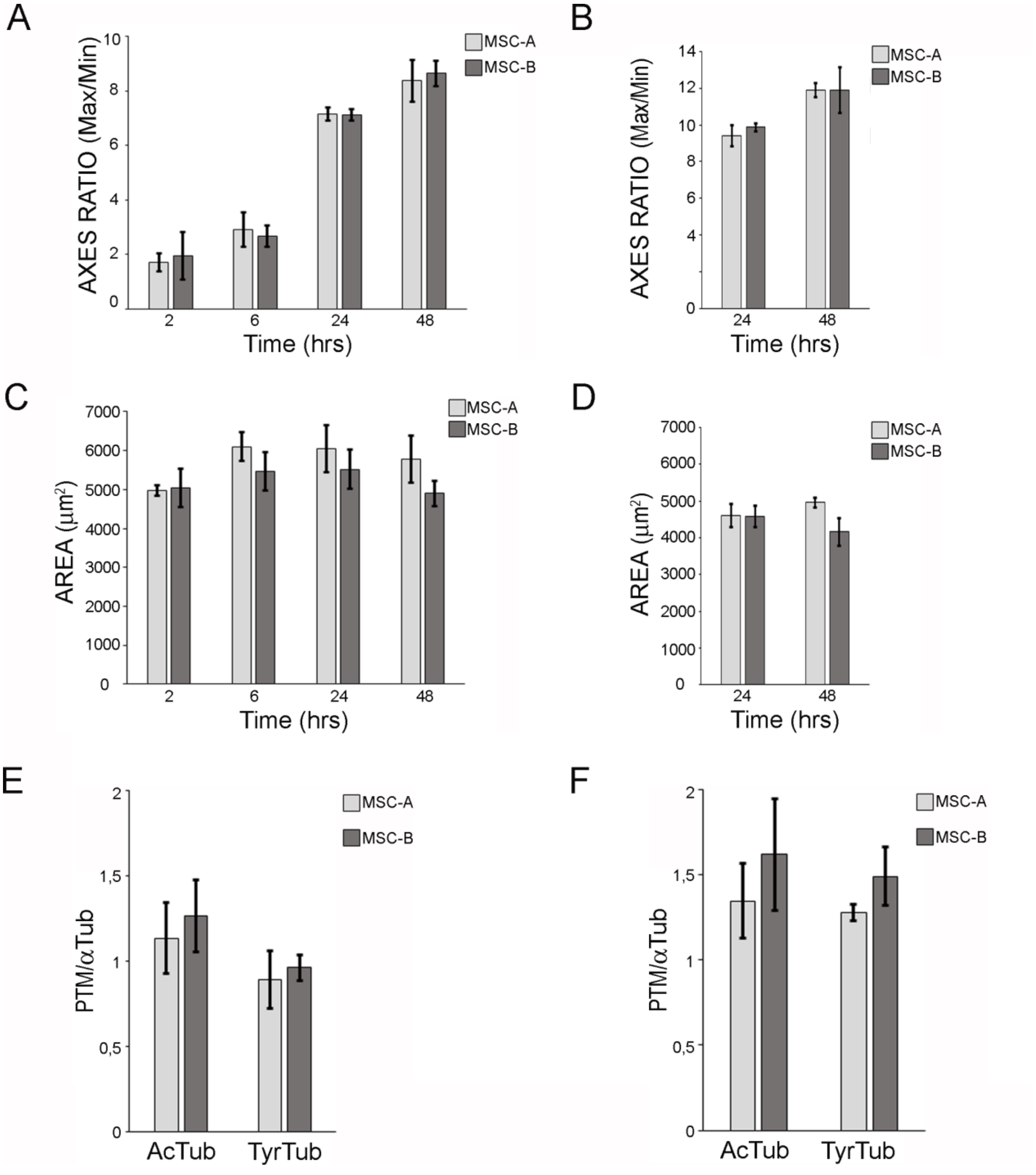

Fig. S4: Comparative analysis of morphology and α-tubulin PTMs in the two subgroups of MSCs from PSP patients

Morphometric analysis showing ratio between maximum and minimum cellular axes (A) or surface area (C) of cells from MSC-A and MSC-B subgroups of PSP patients. In (B) and (D) are reported measures restricted to fibroblastic-like cells at 24 and 48 hours (hrs).

Densitometric analysis of the levels of acetylated (AcTub) and tyrosinated (TyrTub) α-tubulin in whole cell extracts of human MSCs of the two subgroups (i.e. MSC-A and MSC-B) at early (P2, E) or later (P5, F) passages in culture. Values of each α-tubulin PTM were normalized on the level of total α-tubulin of the relative sample. Values are expressed as fold change on control level, as in Fig. 4. All values are expressed as mean ± SEM.
